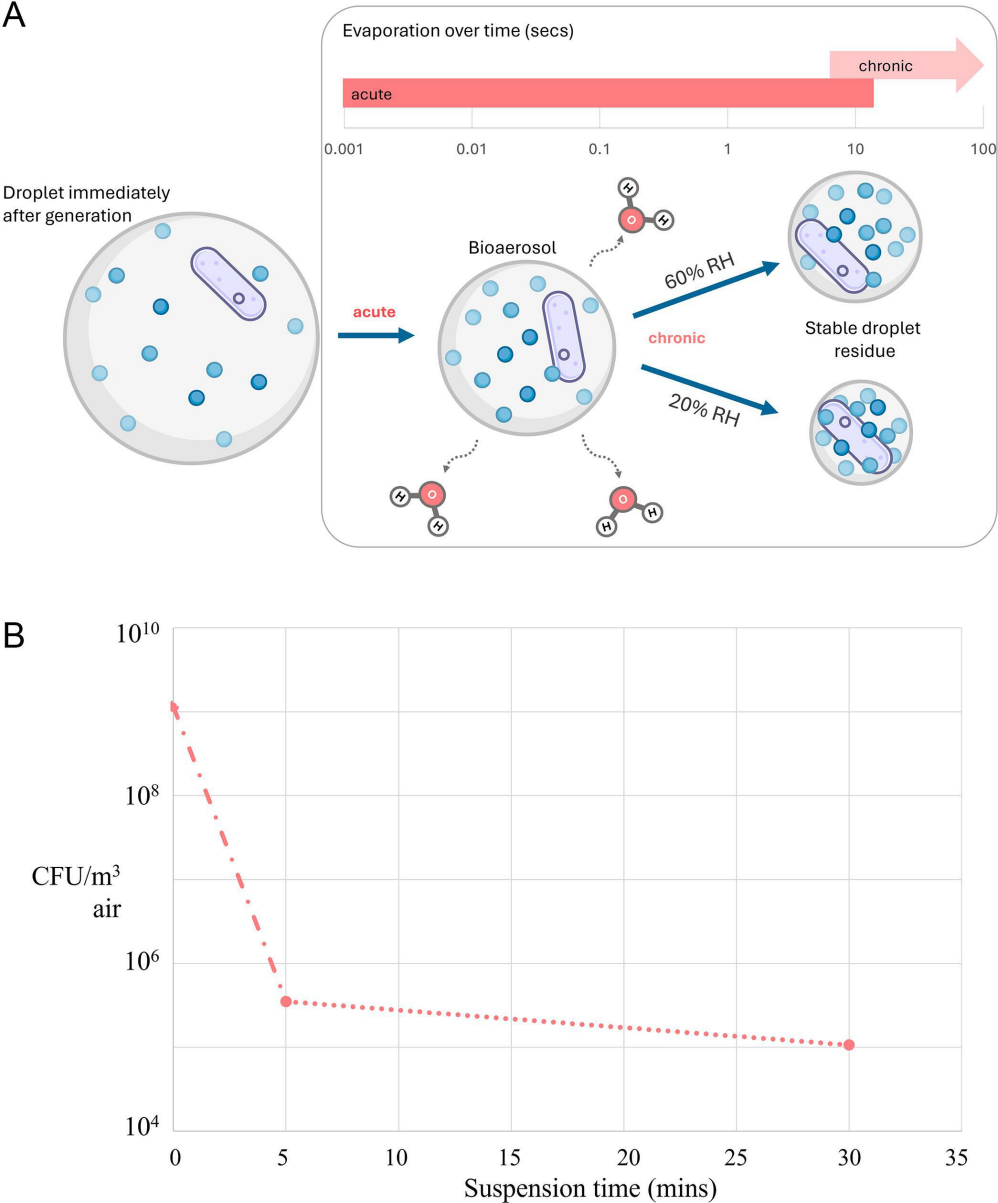# Articles of Significant Interest in This Issue

**DOI:** 10.1128/aem.00972-25

**Published:** 2025-05-21

## A HORSE TALE OF *DIFFICILE* PROPORTIONS

The human pathogen *Clostridioides difficile* is prevalent in feral horses. Hain-Saunders et al. (e02114-24) characterize isolates from horse feces, providing a baseline for evaluating the effects of domestication on equine *C. difficile* transmission and infection.


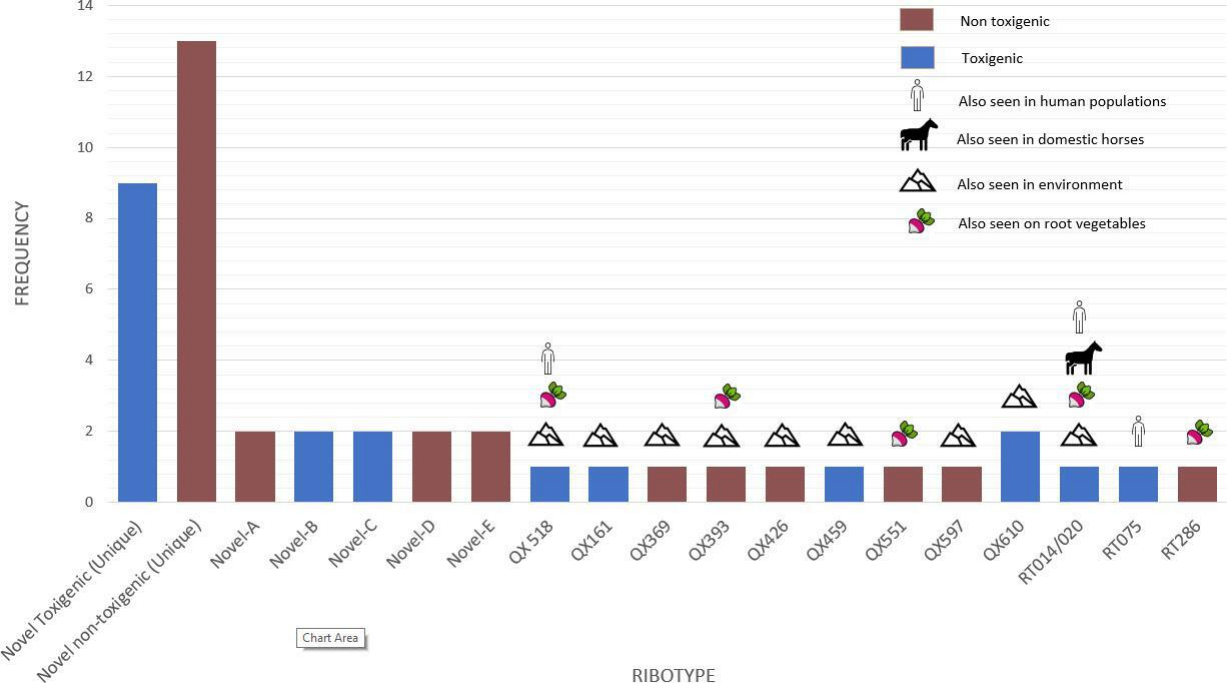


## THE CABLE BACTERIA FAMILY ADDS A NEW MEMBER

Cable bacteria form multicellular filaments for electron transport across centimeter-thick sediments. Hiralal et al. (e02502-24) use genetic and morphological characterization to reveal a new marine species with unique adaptations for long-range conduction.


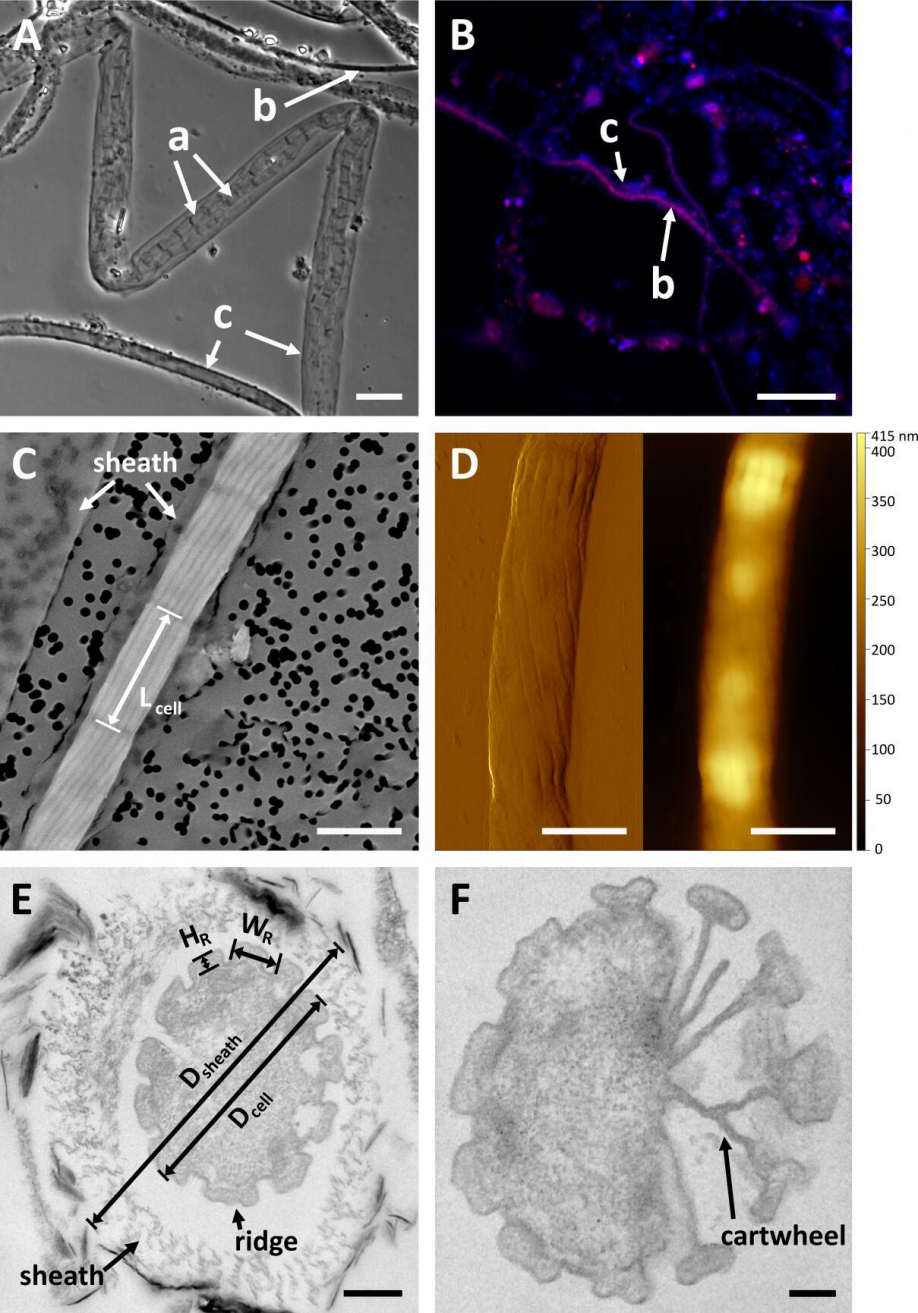


## STRUCTURAL CONTROL OF SYMBIOTIC DEFENSE

Kamp and colleagues (e02163-24) employ multidimensional *in situ* imaging techniques to show how the structure of the reproductive organ in the female bobtail squid controls colonization by beneficial bacteria and deploys them for egg defense.


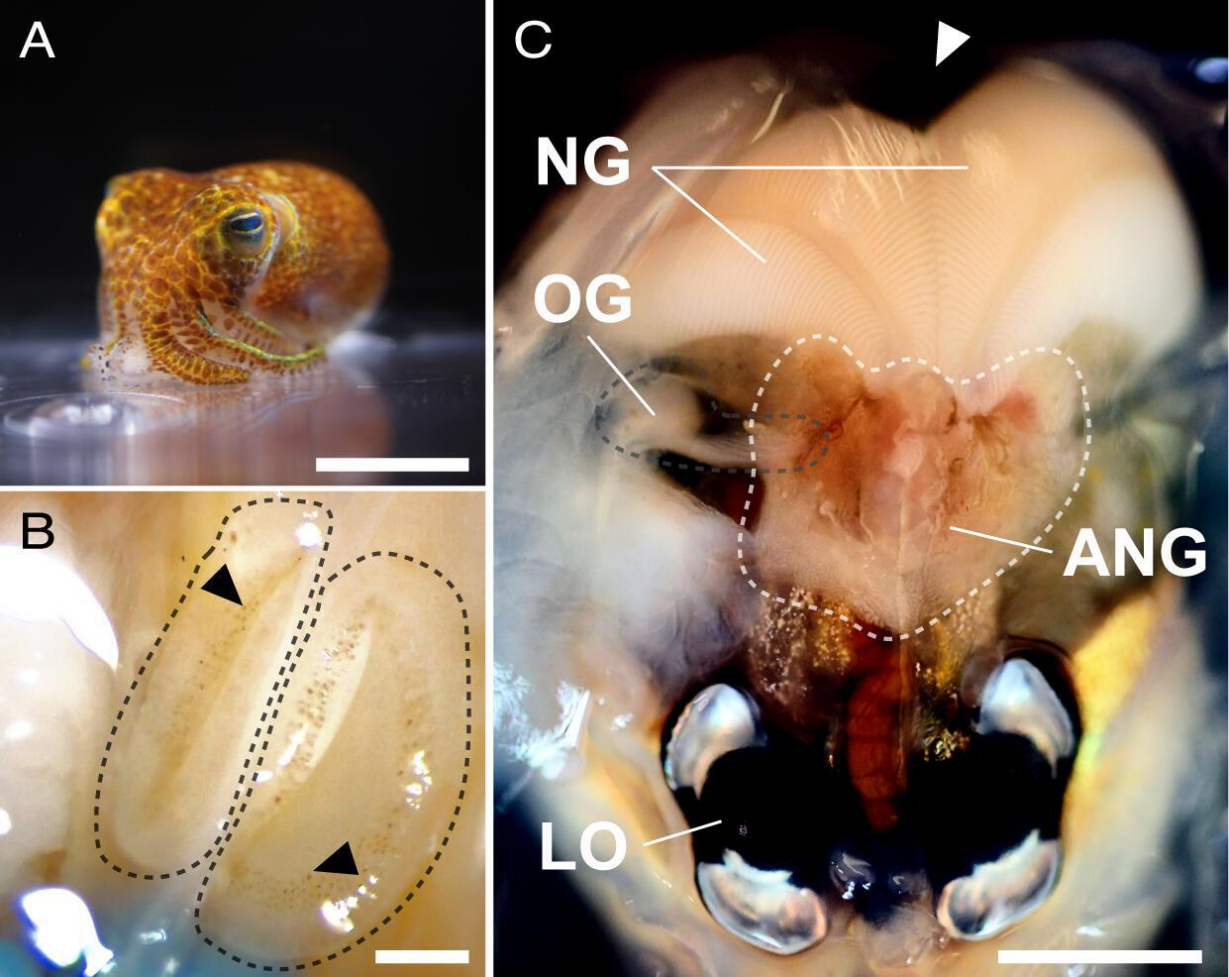


## A SAFE APPROACH TO STUDY AVIAN INFLUENZA SPILLOVER INTO CATTLE

Aubrey et al. (e02356-24) show that inactivation of highly pathogenic avian influenza H5N1 from cattle allows for safe transfer of the virus to lower containment laboratory settings for downstream analyses.


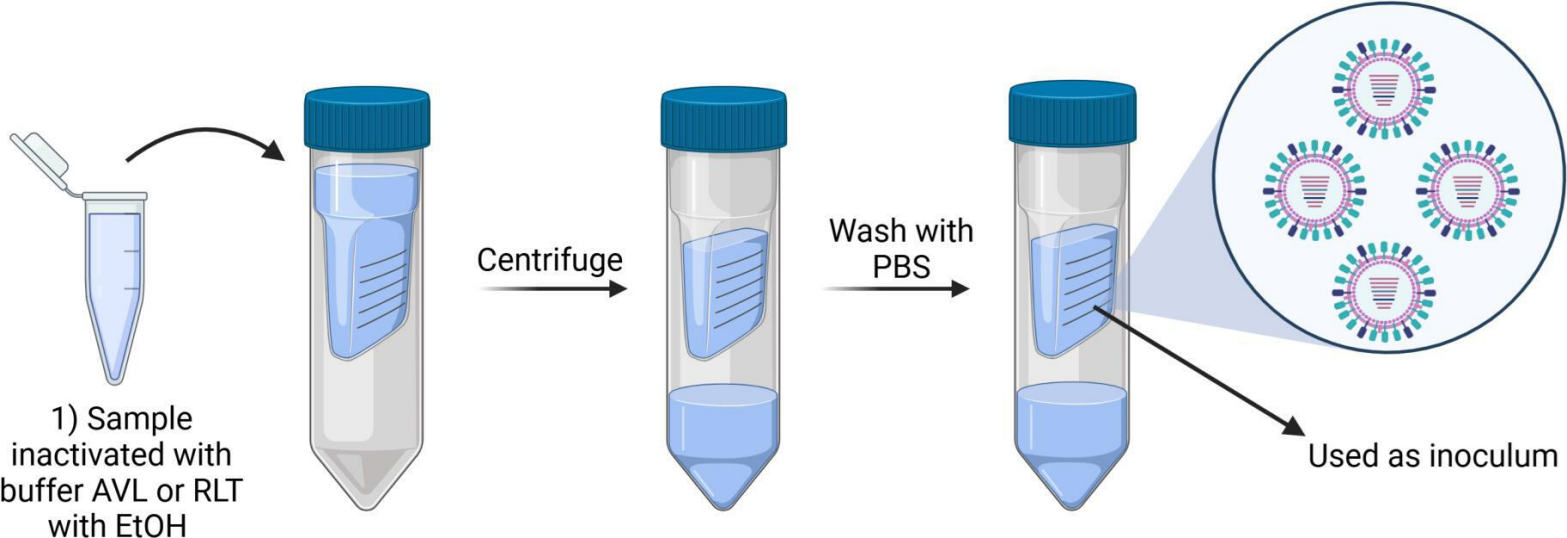


## CrAssphages AS FECAL BIOMARKERS

*Bacteroidetes* bacteriophages named CrAssphages are uniquely abundant in human feces. Their detection in an Amazonian river by Martins et al. (e01470-24) showcases their utility as biomarkers for human fecal pollution in aquatic systems.


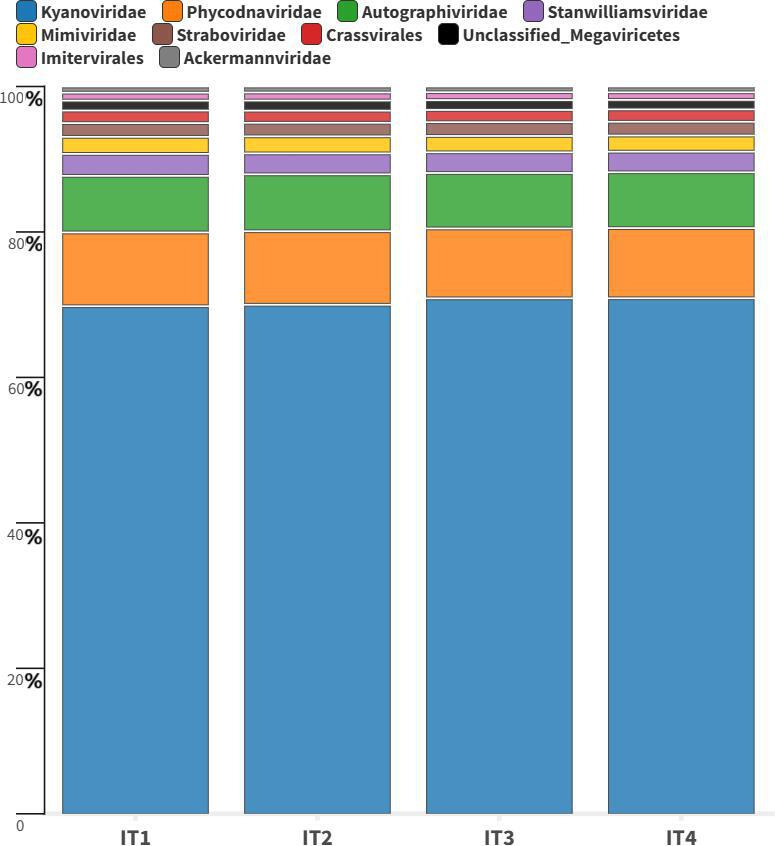


## A SUBSPECIES VIEW OF FOOD SAFETY

This minireview by Flörl et al. (e00524-25) examines the need for characterizing subspecies variation in food microbiomes and integrating strain-level characterization with community-level studies. This is particularly important for assessing food quality and safety.


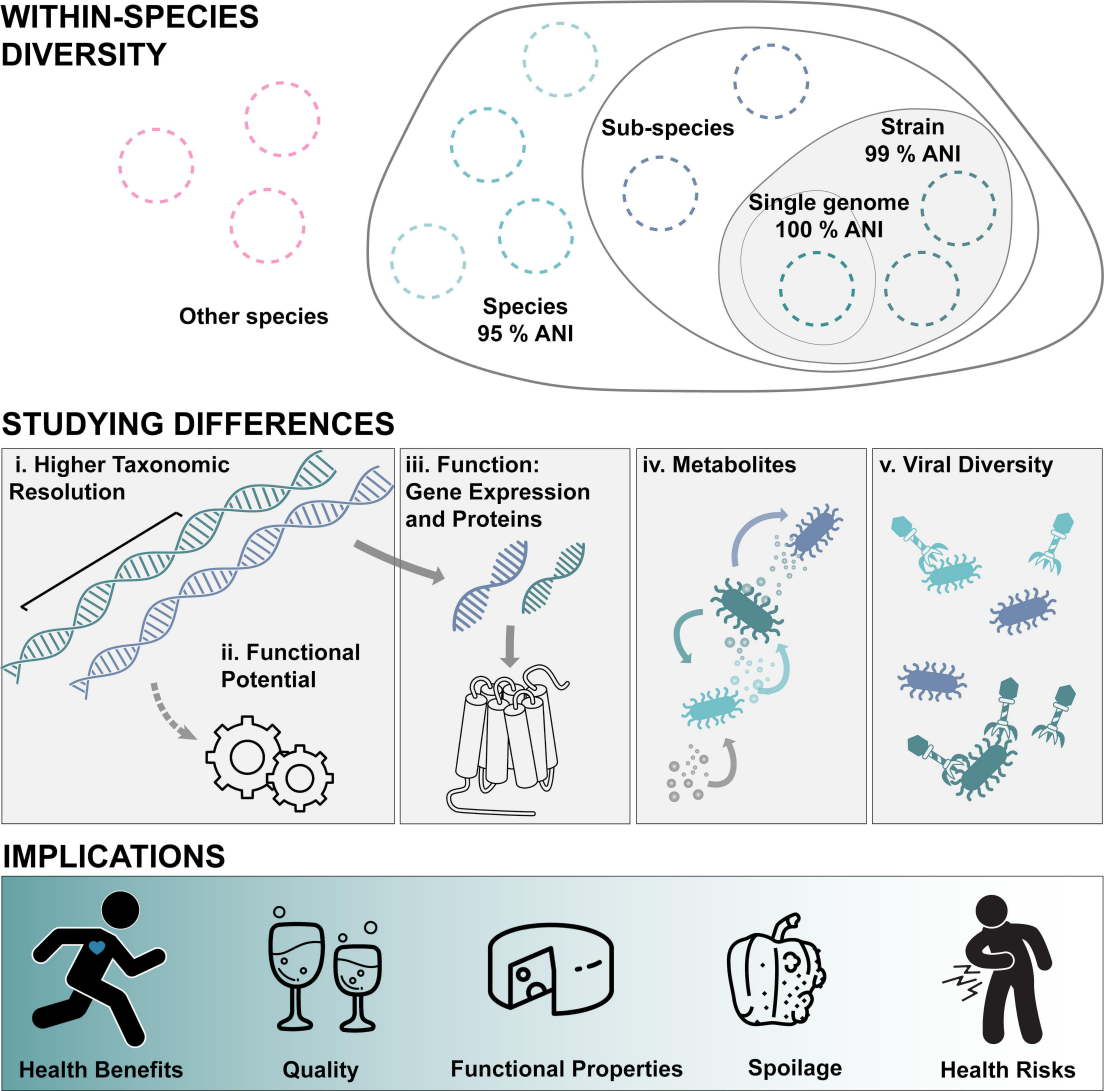


## BIODEGRADATION GETS OILY

Chemicals commonly used to treat oil spills can be enriched for microbial taxa that mineralize the oil dispersants into CO_2_. This study by Lech et al. (e02334-24) informs oil spill responders of treatment options that are both effective and sustainable.


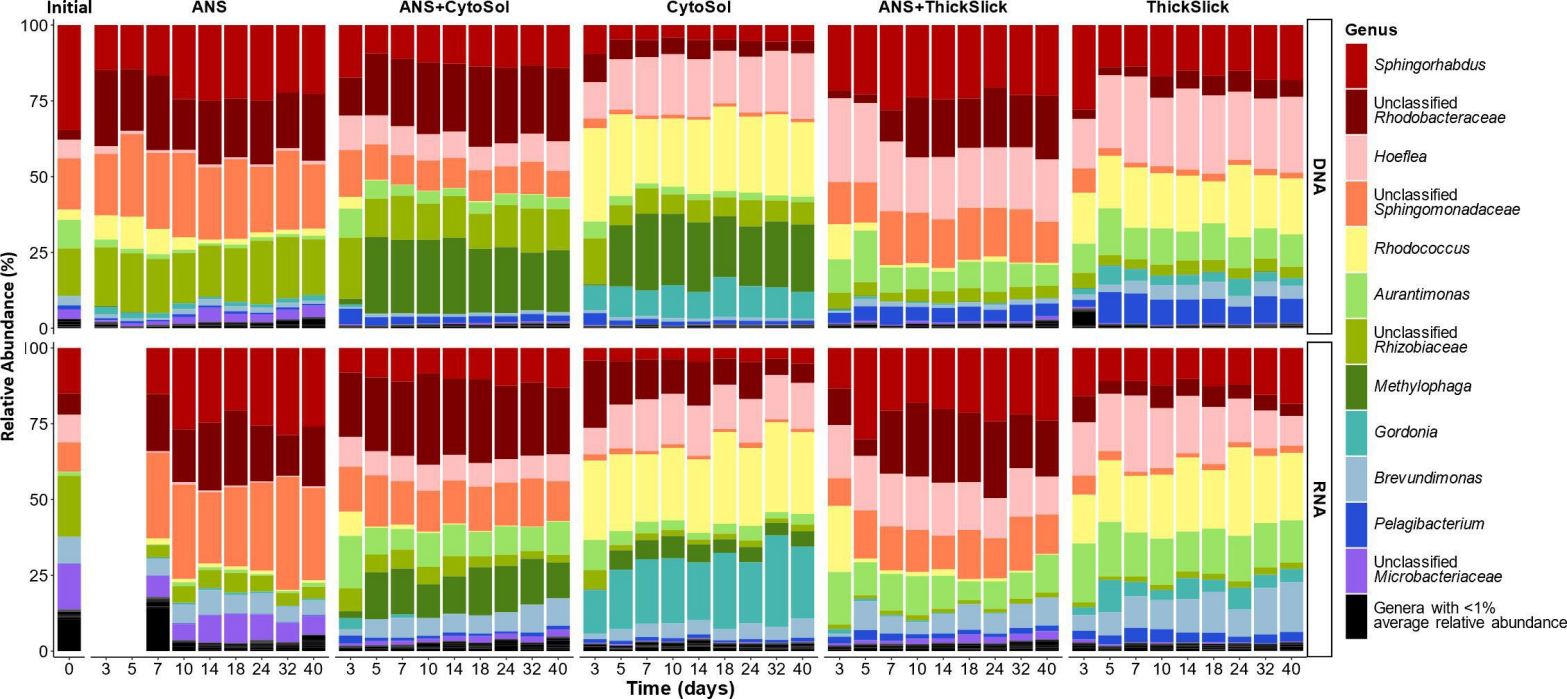


## AEROBIOLOGY UNDER REVIEW

Kraus et al. (e00148-25) present a much-needed review of the methods and improvements in profiling bioaerosol activity to better understand the ecophysiology of the aerobiome and control of live bioaerosols.